# Efficacy of Dupilumab in a Young Woman with Refractory Cutaneous Lichen Planus: A Case-Based Review

**DOI:** 10.3390/diseases13070225

**Published:** 2025-07-18

**Authors:** Cristina Guerriero, Luisa Boeti, Francesco Mastellone, Giulia Coscarella, Gennaro Marco Falco, Gerardo Palmisano, Helena Pelanda, Ketty Peris, Donato Rigante

**Affiliations:** 1 Unit of Dermatology, Dipartimento Scienze Mediche e Chirurgiche, Fondazione Policlinico Universitario A.Gemelli IRCCS, 00168 Rome, Italy; cristina.guerriero@policlinicogemelli.it (C.G.); boetiluisa@gmail.com (L.B.);; 2 Dipartimento Universitario di Medicina e Chirurgia Traslazionale, Università Cattolica Sacro Cuore, 00168 Rome, Italy; 3 Department of Life Sciences and Public Health, Fondazione Policlinico Universitario A. Gemelli IRCCS, 00168 Rome, Italy; 4 Rare Diseases and Periodic Fever Research Center, Università Cattolica Sacro Cuore, 00168 Rome, Italy

**Keywords:** cutaneous lichen planus, dupilumab, atopic dermatitis, pruritus, personalized treatment, innovative biotechnologies

## Abstract

*Background:* Cutaneous lichen planus (CLP) is a chronic inflammatory T cell-mediated disease driven by a mixed Th1 and Th2 lymphocyte population, for which many of the currently available treatments have poor efficacy. *Aim:* The aim of this study was to indicate the clinical success of dupilumab administration after two years of treatment in a case of longstanding CLP and to perform a review of the medical literature related to the use of dupilumab in different dermatologic settings and in CLP. *Case presentation:* One 26-year-old woman with a previous history of atopic dermatitis had a long-lasting skin condition, referred to as a suspected lichen, which started when she was 7 years old. Her disease exhibited a relapsing–remitting course with severe bouts of pruritus over a very long period. The final histological diagnosis of CLP was confirmed at the age of 26. Starting dupilumab (injected subcutaneously at a dose of 600 mg followed by a maintenance dose of 300 mg every two weeks) resolved the skin scenery of this patient, who is currently in full remission. *Conclusions:* The remarkable recovery from CLP obtained via treatment with dupilumab in this single-patient case study emphasizes the potential therapeutic implications of targeting the Th2 pathway in this skin disorder.

## 1. Introduction

Cutaneous lichen planus (CLP) is a chronic inflammatory disease of the skin, hair, and nails characterized by a mysterious pathogenesis which is potentially caused by infectious, genetic, and immune dysregulation mechanisms [1]: it affects less than 2% of the overall population and may manifest itself in heterogeneous forms: atrophic, hypertrophic, actinic, pigmentosus, ulcerative, and generalized, in all age groups, regardless of ethnicity. The medical literature examining treatment modalities for this condition in relation to patients’ sociodemographic, clinical, and labwork characteristics is rather limited: the pathological underlayer of CLP still needs to be elucidated, as shown by the inadequacy of different therapies [2]. Its ‘classic’ presentation includes red-to-brown or violaceous polygonal papules and plaques that are flat-topped, slightly scaly, and intensely pruritic; the localization is characteristically symmetrical and involves the extremities with flexural surfaces on the forearms, wrists, ankles, and the dorsal surfaces of the hands and shins [3,4,5]. Most cases of CLP resolve spontaneously within a few years, and the objective of available treatments, according to a small number of studies and anecdotal reports, is to achieve their complete resolution quickly and to manage the severity of itching [6]. Topical corticosteroids and tacrolimus are the usual first-line treatments offered to patients with CLP, but different immunosuppressants could be considered in refractory forms or in the most severe generalized cases [7].

## 2. Key Information About the Pathogenesis of Cutaneous Lichen Planus

The central phenomenon underlying the development of CLP is the cell-mediated immune response among basal keratinocytes, which subsequently become a source of antigens that trigger inflammation: the pathogenesis is likely founded on T cell-mediated immunity against either endogenous or exogenous antigens, but even though it is commonly recognized as a T cell-induced inflammatory condition, the specific immune cell populations and pathogenic cytokines driving the onset of CLP have not been unambiguously clarified [8]. The main characters involved in CLP are helper T cells, cytotoxic T cells, natural killer cells, antigen-presenting cells such as dendritic cells, and keratinocytes. The most important role is attributed to CD8+ T cells, which infiltrate the epithelia and lead to apoptosis of the basal-layer keratinocytes. Several studies have demonstrated high CD4^+^ and CD8^+^ cell populations in the skin of CLP patients along with a strong Th1 and interferon (IFN)-γ signature [9]. However, even a Th2 cell lymphocyte-driven response may crucially result in CLP followed by increased production of interleukin (IL)-4, IL-13, and IL-10 [10].

CLP onset depends on damage-associated molecular patterns such as S100A8/A9 stimulating Toll-like receptors and triggering IFN oversecretion from plasmacytoid dendritic cells: IFN upregulates further chemokines, such as CXCL9 and CXCL10, activating inflammatory dermal dendritic cells to interact with naïve T lymphocytes and promote both the differentiation and expansion of Th1 helper and cytotoxic cell subpopulations [11]. Tissue-resident memory T lymphocytes respond via the plentiful production of IFN-γ, tumor necrosis factor (TNF)-α, IL-2, IL-17, and IL-22: this cascade supports Th1- and Th17-related pathways, reinforcing the vicious circle of inflammation and the production of reactive oxygen species, inducing both profound epidermal damage and keratinocyte apoptosis [12]. The widespread cutaneous lesions in patients with generalized CLP, who are frequently resistant to first-line treatment, suggest a more severe inflammatory process in this subgroup.

Recently, an association between CLP and the risk of cardiovascular diseases has been hypothesized. In fact, higher fasting blood glucose and higher lipid levels have been reported in patients with CLP [13,14]. Moreover, Daye et al. emphasized the relationship between oral forms of lichen planus and metabolic syndrome, which was found in 60% of cases characterized by the most relevant severity [15]. Other recent studies attributed a role to oxidative stress in the pathogenesis of different skin diseases with an autoimmune component, such as alopecia areata, pemphigus vulgaris, psoriasis, and vitiligo: disrupted oxidative stress has been also suggested in patients with CLP, who can display an imbalance between oxidants and antioxidants, leading to altered thiol-disulfide homeostasis at the skin level [16]. 

## 3. A Dual Involvement of Infectious and Genetic Mechanisms in Cutaneous Lichen Planus

Since the clinical history of ‘classic’ CLP is characterized by acute onset with a self-limiting course, an infectious etiology has been often suspected to be involved as a potential disease primer. For instance, hepatitis C virus (HCV) infection and CLP have been correlated, and it was recommended that a serology test for HCV should be performed in patients with CLP [17]: HCV is up to thirteen times more commonly detected in CLP patients, and this relationship was explored by numerous studies and meta-analyses conducted during the last few decades [18]. HCV components may probably lead to host immune response dysregulation, as terminally differentiated and virus-specific CD8+ T lymphocytes were discovered in CLP lesions [19]. Similarly, hepatitis B virus (HBV) infection is more frequent in patients with CLP, who become prone to the development of lichenoid disorders with pitted keratolysis following biologic abnormalities that still need to be elucidated [20]. There are also data showing that CLP might be a complication observed in children receiving the HBV vaccine or also other vaccinations, as shown by anecdotal reports [21]. Furthermore, an increased prevalence of human papillomavirus has been found in patients with the oral localization of lichen planus [22], while another presumed possible CLP trigger could be Epstein–Barr virus [23]. Among the eventual relationship with bacterial infections causing CLP, *Helicobacter pylori* has been proposed as a contributor, though with conflicting results among different reports [24].

The genetic predisposition to CLP was first suspected after revealing this disease in identical twins [25]. Such an observation confirmed the existence of a familial lichen planus form, which constitutes up to 10% of all CLP cases and is characterized by earlier onset, the risk of frequent relapses, a higher possibility of treatment resistance, and higher rates of oral mucosa involvement [26]. Furthermore, HLA-based susceptibility association studies have identified a significantly raised frequency of HLA-A3 in CLP [27]. More detailed data on genetics were provided by a phenome-wide association study revealing six single nucleotide polymorphisms highly associated with CLP, among which rs1794275 in the *HLA-DQB1* and *HLA-DQA2* genes was the most significant [28]. Another genome-wide association study identified two single nucleotide polymorphisms, i.e., rs884000 in the *NRP2* locus and rs538399 in the *IGFBP4* locus, associated with CLP [29].

Various drugs, especially those from the group of antihypertensives and biological agents such as TNF antagonists, have been reported to induce lichenoid skin reactions [30,31].

The very recent advance in dermatopathology has introduced a deeper understanding of immunologic backgrounds behind many skin disorders and a concurrent progress of therapies characterized by enhanced precision and efficiency. All available systemic therapies for different dermatoses, for instance atopic dermatosis (AD), were primarily given per os such as cyclosporine, methotrexate, azathioprine, and mycophenolate mofetil, which modulated the underlying pathophysiologic pathways of multi-faceted cutaneous diseases. However, based on the available data and more recent experience, some biological drugs have offered more effective disease control with overall favorable safety profiles in comparison with the available systemic treatments [32]. We share our experience related to a young woman who was diagnosed with CLP and who finally showed a significant clinical response after starting biological therapy with dupilumab.

## 4. Case Presentation

A 26-year-old woman presented to our Dermatology Unit at the Fondazione Policlinico Universitario A. Gemelli IRCCS, Rome, complaining of chronic erythematous papules on both feet, ankles, and legs as well as her abdomen, which was accompanied by a burning sensation and severe pruritus; she also had pruritus in the genital area. A history of AD combined with skin lesions on the feet that first appeared at the age of 7 years was also reported: this condition exhibited a relapsing–remitting course over a long period, which tended to worsen during summer or during stressful events and was referred to as suspected CLP. The patient had been previously treated with multiple cycles of antihistamines and prednisone, tapered and suspended or resumed for different months combined with topical corticosteroids, which led to a temporary and only partial improvement of skin lesions. Some areas of residual skin lichenification and xerosis were sometimes appreciated with an occasional reduction in pruritus. Allergy tests were negative with the exception of patch tests revealing allergy to nickel and tixocortol. Numerous episodes of idiopathic Raynaud’s phenomenon after cold exposure were also reported.

At 26 years, the patient had a severe intensification of her skin disease, involving feet and hands, with highly pruritic papules which also bled if she was sweating: for this reason, she requested our clinical assessment. Her current itch symptoms were unresponsive to oral antihistamines and significantly interfered with her sleep and daily activities with a numerical rating scale (NRS) of 10/10 (with 10 being the worst pruritus possible). The patient was a random smoker and reported no alcohol or substance use; she also reported no sick contacts. The patient’s family history was notable for psoriasis in her mother and diabetes and psoriatic arthritis in the maternal grandfather.

**Figure 1:**
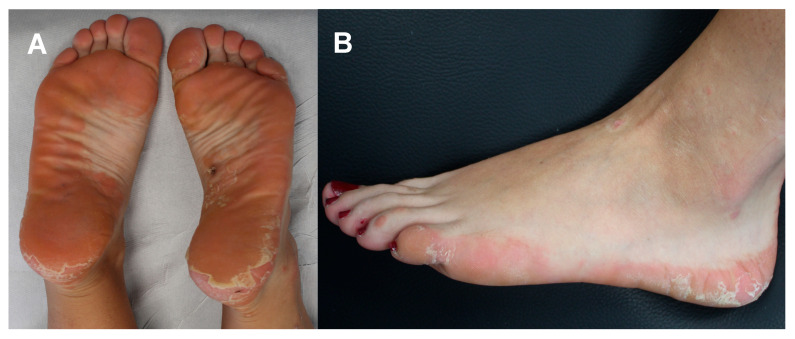
Pretreatment clinical assessment of the patient: erythematous scaly plaques with well-defined borders located on the soles of feet (**A**) and well-defined flaky plaques extending from the heel to the lateral region of foot (**B**).

On physical examination, the patient was afebrile with a normal heart rate, normal blood pressure, normal oxygen saturation while breathing ambient air; the abdomen was normal to palpation with no rebound tenderness, guarding, palpable masses, or hepatosplenomegaly. Capillary refill was normal, and there was no palpable lymphadenopathy. No further signs of malar rash or abnormalities in the finger and toe extremities were noted. Neurologic examination, including assessment of cranial nerves, strength and sensation, was normal. The body mass index (the weight in kilograms divided by the square of the height in meters) was 20.07. Upon specific dermatological examination, a few small red papules were seen on the ankles and Achilles tendons, but mostly erythematous scaly plaques with well-defined borders on the soles of feet were evident with well-defined flaky plaques extending from the sole to the lateral regions of both feet (see Figure 1). Wickham’s striae were not visualized at the dermoscopy. No associated nail, hair, or mucosal abnormalities were noted.

**Figure 2:**
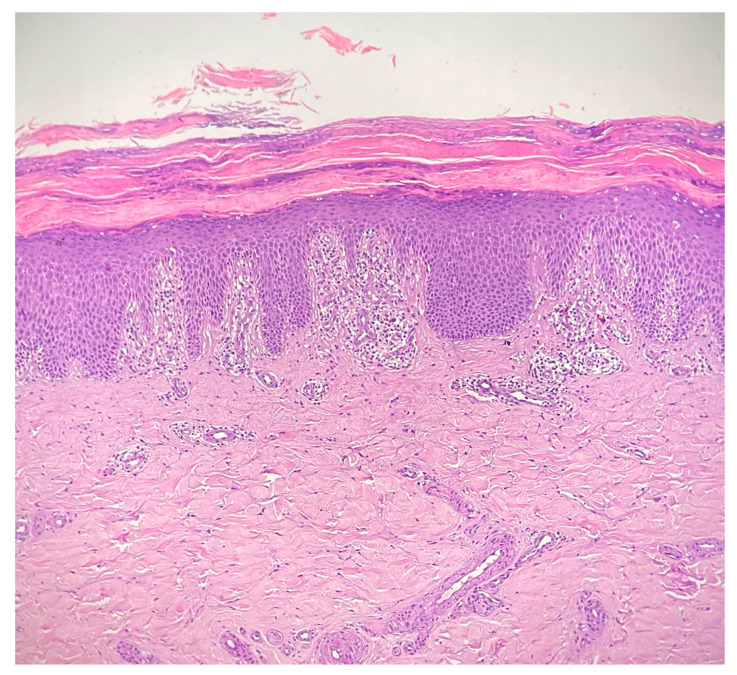
Interface dermatitis showing a degeneration of the basal layer of the epidermis with vacuolopathy, spongiosis and hyperkeratosis, that focally blurs the dermal–epidermal junction, consistent with the diagnosis of cutaneous lichen planus; the inflammatory infiltrate is chiefly represented by lymphocytes and forms a band-like arrangement along the dermal–epidermal junction (hematoxylin and eosin staining 20×).

Complete blood count, liver and renal function tests as well as the complete autoimmunity panel and thyroid function tests were within normal limits. In particular, the anti-nuclear antibody, anti-double-stranded DNA antibody, anti-Scl-70 antibody, anti-neutrophil cytoplasmic antibody, and rheumatoid factors were negative. The erythrocyte sedimentation rate and complement C3 and C4 levels were normal; a screening test for hepatitis viruses was negative as well as the screening for malignancies. Skin biopsy of the sole of patient’s left foot showed diffuse basal vacuolopathy with spongiosis and hyperkeratosis that focally blurred the dermal–epidermal junction, which was consistent with the diagnosis of CLP (see Figure 2). Unfortunately, additional specific data or labwork investigations related to the patient were not available.

**Figure 3:**
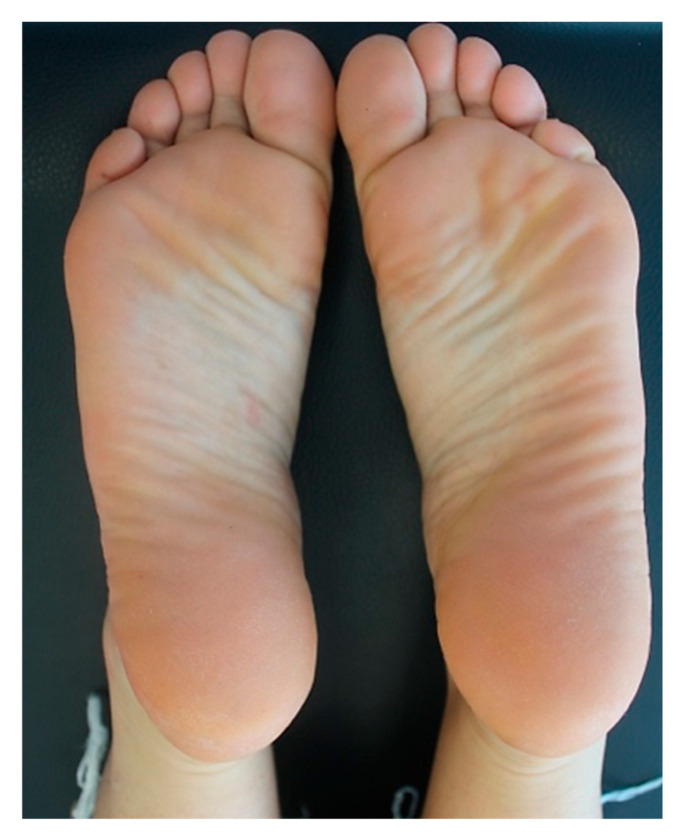
Post-treatment clinical assessment of the patient: resolution of the erythematous scaly plaques after twelve weeks of treatment with dupilumab.

Due to the past history of AD and with the aim of controlling the disabling pruritus associated with CLP, the patient underwent a 3-month treatment with full-dose cyclosporine (200 mg/day), which was then tapered and stopped because of limited efficacy. Afterwards, she was started on subcutaneous injections of dupilumab at a loading dose of 600 mg, which was followed by the maintenance dose of 300 mg every two weeks. At the 5-week follow-up, the patient dramatically showed a substantial improvement of CLP lesions with total regression of the plantar lesions alongside the reduction in pruritus and genital burning (NRS pruritus 2/10). At the 5-month follow-up visit, the patient had no lesions with complete regression of plantar lesions (NRS pruritus: 0/10). Figure 3 shows the resolution of the erythematous scaly plaques on both feet after twelve weeks of dupilumab. At the 2-year follow-up, the patient is still on a monthly dose of dupilumab, given since the last month, and is completely symptomless; her overall skin remains normal with no reported adverse events related to the ongoing dupilumab therapy.

## 5. The Application of Biological Agents and Dupilumab in Protean Dermatological Landscapes

Many specific mediators of inflammation have become important targets in the dermatologists’ therapeutic armamentarium, inflecting the immune system through either stimulatory or inhibitory actions. The oldest agents belonging to this category of drugs act against TNF-α [33]. TNF-α is involved in the recruitment of immune cells to the cutaneous micro-environment, inducing IFN-γ, which in turn augments IL-12 secretion and ultimately causes a Th1 response characterized by an intense phagocytic activity [34]. The TNF inhibitors infliximab, adalimumab and etanercept have been used in various monogenic and complex genomic conditions like autoinflammatory disorders, which are characterized by the recurrence of seemingly unprovoked febrile attacks of variable duration and multi-district inflammation showing even dramatic severity [35,36]. The wide framework of such disorders encompasses a prominent involvement of skin, and different activity scoring systems including skin assessment have been developed [37,38], with cryopyrin-associated periodic syndrome and TNF receptor-associated periodic syndrome displaying the most severe cutaneous manifestations which can be highly responsive to TNF antagonists [39,40]. Concurrently, TNF-blocking agents are largely used in many dermatological conditions, although only a few uses have been approved by drug authorities, including routine use for the treatment of moderate to severe plaque psoriasis and hidradenitis suppurativa; they remain “off-label” for the management of pyoderma gangrenosum, Behçet’s disease, Sweet’s syndrome, sarcoidosis, autoimmune bullous diseases, systemic lupus erythematosus, and systemic sclerosis.

The most important skin disorder successfully treated with biological therapies is AD, which is a chronic inflammatory disease frequently combined with a personal or family history of asthma and allergic rhinitis. It has been considered as a T cell-mediated disorder with a robust cytokine signature with many unmet medical needs at both the diagnostic and therapeutic level: during the past decade, extraordinary progress has been made in the understanding of its immunopathogenesis [41]. In particular, AD has been associated with activation of specific groups of cytokine genes including IL-3, IL-4, IL-5 and IL-13, and TNF-targeted therapies have been employed with variable success in patients with AD [42]. Our comprehension of the inner mechanisms behind AD has led to the discovery that a complex interaction of environmental and genetic factors related to the Th2 pathway, Th1 and Th17 axes, epidermal barrier dysfunction, and JAK/STAT signaling are intricately involved [43]. In detail, AD results from Th2 inflammation mediated by both IL-4 and IL-13, and dupilumab shows the greatest inhibition of Th2 inflammation and impressive power in controlling AD-related pruritus. In fact, the number of AD patients who were successfully treated with this agent has grown substantially over time [44].

More specifically, dupilumab is a fully human monoclonal IgG_4_ antibody that blocks the alpha subunit of the IL-4 receptor and modulates the Th2 immune response via dual inhibition of IL-4 and IL-13: furthermore, this molecule induces a downregulation of Th2 cell activation by blocking the IL-4/IL-13 pathway and leads to Th1/Th2 imbalance, causing a shift toward a Th1-mediated immune response [45]. For this reason, dupilumab—subcutaneously given at the dose of 600 mg and continuing doses of 300 mg every other week—is Food and Drug Administration-approved (since 2017) for the treatment of moderate to severe AD and is also approved as an add-on maintenance therapy in AD patients with eosinophilic phenotype or with oral corticosteroid-dependent asthma, chronic rhinosinusitis with nasal polyps, prurigo nodularis, and eosinophilic esophagitis [46]. Adult and adolescent patients with moderate to severe AD followed up for up to 3 years after the initiation of dupilumab experienced sustained and substantial improvement in pruritus, and dupilumab is a promising tool in the treatment of non-atopic pruritus [47]. In fact, idiopathic chronic pruritus—an example of how mammals use sensory responses to remove noxious stimuli—may result in refractory to multiple treatment regimens including topical and systemic corticosteroids, antihistamines, antidepressants, antiemetics, opioid-antagonists, cannabinoids and even anticonvulsants. Indeed, Th2-mediated inflammation is crucial in chronic pruritus, starting from complex interactions between neurons, keratinocytes and immune cells via chemokines, neuropeptides, alarmins, IL-4, and IL-13, though it is still uncertain whether these proinflammatory mediators can be considered pruritogens [48]. Several studies have explored the potential therapeutic benefit of dupilumab in different forms of epidermolysis bullosa, improving itch and blistering [49]. Some patients with chronic urticaria, diagnosed without any identified trigger, whose pruritus is highly debilitating and recalcitrant to standard treatments (antihistamines or even omalizumab and/or cyclosporine) have been successfully treated with dupilumab [50]. Dupilumab has also been useful in the treatment of dermatoses with sub- or intra-epidermal blistering, like bullous pemphigoid caused by autoantibodies against the proteins BP180 and BP230 of hemidesmosomes, confirming that IL-4 and IL-13 as well as other Th2-related cytokines are involved in its pathogenesis [51]. Several case reports and case series have also shown the clinical efficacy of dupilumab in patients with alopecia areata, including those with alopecia totalis and universalis, who failed to respond to multiple previous treatments [52]. After extensive ineffective therapies, dupilumab may consistently reduce itch in patients with dystrophic epidermolysis bullosa and Hailey–Hailey disease, which are caused by the aberrant development of desmosomes within epidermis and subsequent acantholysis, as well as in patients with Netherton syndrome, which is a rare inborn error of immunity with the phenotypic triad of trichorrhexis invaginata, congenital ichthyosiform erythroderma, and multiple atopic manifestations [53]. Dupilumab has been also effective in cases of cold urticaria, exercise-induced cholinergic urticaria, palmo-plantar pustulosis, hidradenitis suppurativa, and graft-versus-host disease (after hematopoietic stem cell transplantation), all sharing similar histologic features with AD [53]. Conversely, cold-induced urticaria-like rashes belonging to the group of cryopyrin-associated periodic syndrome promptly respond to IL-1 blockers, such as anakinra, which is the recombinant form of the IL-1 receptor antagonist [54,55]. The long-term blockade of IL-1 has been found to restore the clinical equilibrium in many systemic inflammasomopathies of childhood, and IL-1 inhibitors have become cardinal weapons in managing both monogenic innate immunity defects and a plethora of polygenic diseases occurring in children, including different skin disorders [56]. Anakinra has also been demonstrated to have activity in the treatment of the non-Langerhans cell histiocytosis known as Erdheim–Chester disease and also in some patients with lichen planus-associated pruritus [57]. Conversely, the allele 2 of the gene *IL1RN*, coding for the IL-receptor antagonist, may be considered as a genetic marker for both disease severity and disease extent in lichen sclerosus [58]. The efficacy of dupilumab has been also proved in chronic photodermatoses such as actinic dermatosis and actinic prurigo, which are clearly induced by exposure to ultraviolet radiation and can resemble photosensitive AD [59]. Hematologic malignancies can be also associated with pruritic rashes with skin eosinophilia, while hyper-eosinophilic syndromes can present with eczema and other organ manifestations, sometimes responding to dupilumab after the failure of systemic corticosteroids [60]. Finally, cutaneous T cell lymphomas, encompassing mycosis fungoides and Sézary syndrome, can be accompanied by refractory pruritus that drastically affects the quality of life of patients, especially in palliative settings: dupilumab has shown reduction in itch as a supportive care treatment in these patients though with contradictory results [61,62]. Table 1 shows a list of skin disorders (different from AD) in which dupilumab has shown notable efficacy.

## 6. Update of Dupilumab Efficacy in Cutaneous Lichen Planus

Novel possible indications for dupilumab are increasing, including nummular eczema, bullous pemphigoid, alopecia areata, and Netherton syndrome as well as respiratory diseases such as allergic bronchopulmonary aspergillosis, chronic eosinophilic pneumonia, and allergic rhinitis [63]. The role of dupilumab in the management of CLP and more specifically of CLP-associated pruritus has not been studied in clinical trials. No perspective multicenter randomized double-blind placebo-controlled trials using alternative drugs to corticosteroids are specifically in progress for CLP. Our report has described a young woman with diffuse skin lesions referred to as CLP and severe localized pruritus who experienced a consistent improvement of all skin lesions and itching following treatment with dupilumab.

CLP is a chronic and relapsing disease involving cytotoxic and mixed helper T lymphocyte populations (Th1 and Th2): its main feature is epidermal hypergranulosis, which manifested as reticulate structures (or Wickham’s striae) on the lesions’ surface [64]. A very recent review of CLP has revealed that patients with generalized disease are often resistant to different lines of treatment and may require alternative therapies such as ultraviolet B phototherapy and methotrexate [65]. However, Khurana et al. found that patients with generalized CLP had a relapse rate of 40.6% among those who initiated treatment with methotrexate [66]. Zhou et al. underscored both the clinical effectiveness and safety of dupilumab for the treatment of CLP in elderly patients who had a higher risk of adverse events if treated with traditional systemic immunosuppressants due to age-related physiologic changes, the use of multiple concomitant drugs, and eventual comorbidities [67].

Anecdotal reports of patients with CLP treated with dupilumab, leading to the resolution of both skin manifestations and pruritus, are related to a 92-year-old woman with a concomitant history of type 2 diabetes and hypertension and to a 52-year-old man: the first was referred to dermatology consultation for widespread CLP and generalized pruritus lasting over two years, which was unresponsive to oral antihistamines and significantly interfering with her sleep, and who responded to dupilumab; the second had a new-onset progressive pruritic CLP on the arms, hands, legs, feet, chest, and back, which responded to dupilumab after the failure of halobetasol and tacrolimus ointments and also after the failure of oral corticosteroids given in the previous 6 months [68,69].

We need also to consider that lichenoid dermatoses have been reported as paradoxical adverse effects while treatment with dupilumab was in progress. In fact, though a quite typical side effect of dupilumab is conjunctivitis, there are many cutaneous reactions which might occur under treatment with dupilumab such as new-onset psoriasis, seborrheic dermatitis, and even CLP. There are recent reports of patients developing psoriasis as a secondary skin condition after treatment with dupilumab for prurigo nodularis, which is probably related to an immune shift from Th2 to a Th1-mediated inflammatory process [70]. Among further unexpected reactions, CLP occurred during treatment with dupilumab for AD in a 60-year-old woman with a previous history of allergy and alopecia areata after the failure of cyclosporine, which was primarily resulting from the upregulation of Th1 chemokines including IFN-γ, TNF, IL-1α, IL-6, and IL-8 [71]. Kern et al. described a 23-year-old woman who had suffered from severe AD since early childhood and was put on dupilumab therapy but developed flat-topped violaceous papules on her wrists, fingers, abdominal wall, upper thighs, and lower legs after 11 months of treatment. A histological examination of skin biopsy showed hyperkeratosis, acanthosis, liquefaction degeneration of the basal cell layer of the epidermis, and a band-like lymphocytic infiltrate in the upper dermis, which was consistent with CLP and disappeared only after dupilumab discontinuation [72].

Several lines of treatment can result effective in CLP—specifically for the classic or generalized CLP. Topical corticosteroids and tacrolimus are usually first-line therapies in CLP, but the number of cases showing poor and unsatisfactory results is relevant. Different treatment modalities have been evaluated in a systematic review considering 16 trials (11 randomized controlled ones) by Atzmony et al [73]. Although the overall quality of evidence was low to moderate in terms of treatment efficacy, acitretin, sulfasalazine, and griseofulvin were associated with higher overall response rates than placebo; conversely, narrowband ultraviolet B phototherapy was more effective than prednisolone in achieving a complete response. In addition, treatment with low-dose methotrexate was effective in obtaining full response rates if compared with oral betamethasone, although not significantly. Lastly, oral psoralen combined with ultraviolet A phototherapy had comparable efficacy to narrowband ultraviolet B phototherapy in patients with CLP enrolled in nonrandomized trials [73]. Further large-scale randomized controlled trials are warranted to investigate the efficacy of so many therapeutic approaches, and more investigations are needed to unravel the enigmas of CLP inflammation, explain the nature of its immune dysregulation, ultimately personalize treatment strategies. Considering the therapeutic resistance of many forms of CLP, to elucidate its immune-pathogenetic mechanisms will probably contribute to implement targeted therapies that could reduce prurigo and mitigate the disease’s negative impact on each patient’s quality of life.

## 7. Conclusions

Refractory forms of CLP remain difficult to manage for both the patient and the clinician; in addition, current therapeutic regimens are derived from small uncontrolled studies and case reports. Although primarily considered a Th1-mediated disease, the interplay of various alternative signaling pathways has been suggested in CLP. The specific immune cell populations and pathogenic cytokines driving the development of this disease have not been yet fully clarified: however, many studies support a primary Th1-reflected activation with the differentiation and migration of cytotoxic CD8 T cells, leading to the destruction of epidermal basal keratinocytes. A subsequent proinflammatory cytokine network should promote the overactivity of IL-4, an essential cytokine in Th2 cell differentiation, and enhance production of the downstream cytokine IL-13. The efficacy of treatment with dupilumab, a monoclonal antibody that targets the IL-4 receptor alpha found on T cells, inhibiting both IL-4 and IL-13 signaling, should confirm that CLP derives from a mixed Th1 and Th2 lymphocyte repertoire and that clinical success may be steered by dupilumab with an overall very good safety profile. Our findings in this case of a young woman with CLP that responded to dupilumab, though limited to a single report, suggest that dupilumab interacts with the pathogenic cascade of this disorder, potentially implicating the role of Th2 cells in its pathophysiology. Further data from perspective clinical trials are needed to prove the optimal therapeutic approach for the different clinical phenotypes of CLP.

## Figures and Tables

**Table 1:** List of skin disorders, different from atopic dermatitis, in which treatment with dupilumab has given clinical efficacy.

Skin Disorder	Reference	Type of Study
Epidermolysis bullosa	[49]	Case series
Chronic urticaria	[50]	Couple of cases
Bullous pemphigoid	[51]	Narrative review
Alopecia areata (totalis/universalis)	[52]	Case report
Dystrophic epidermolysis bullosa	[53]	Systematic review
Hailey–Hailey disease	[53]	Systematic review
Netherton syndrome	[53]	Systematic review
Cold urticaria	[53]	Systematic review
Exercise-induced cholinergic urticaria	[53]	Systematic review
Palmo-plantar pustulosis	[53]	Systematic review
Hidradenitis suppurativa	[53]	Systematic review
Actinic dermatosis/actinic prurigo	[59]	Case series
Hyper-eosinophilic syndrome	[60]	Case report
